# Pediatric ping-pong skull fractures treated with vacuum-assisted elevation

**DOI:** 10.1007/s00381-024-06307-w

**Published:** 2024-02-27

**Authors:** Syed D. Ahmed, Virginia D. Allhusen, Michael G. Muhonen, Suresh N. Magge

**Affiliations:** 1 Division of Neurosurgery, Children’s Hospital of Orange County (CHOC), CHOC Neuroscience Institute, Orange, CA USA; 2 Department of Neurosurgery, University of California Irvine Medical Center, Irvine, CA USA; 3 Department of Neurosurgery, University of Michigan, Ann Arbor, MI USA

**Keywords:** Depressed skull fracture, Suction elevation

## Abstract

**Purpose:**

Depressed (“ping-pong”) skull fractures can be treated by different means, including observation, non-surgical treatments, or surgical intervention. The authors describe their experience with vacuum-assisted elevation of ping-pong skull fractures and evaluate variables associated with surgical outcomes.

**Methods:**

The authors present a retrospective review of all ping-pong skull fractures treated with vacuum-assisted elevation at the Children’s Hospital of Orange County in 2021–2022. Variables included patient age, mechanism of injury, fracture depth, bone thickness at the fracture site, and degree of elevation.

**Results:**

Seven patients underwent vacuum-assisted elevation of ping-pong fractures at the bedside without the use of anesthesia. Fractures caused by birth-related trauma were deeper than those caused by falls (*p* < 0.001). There was no significant difference between groups in bone thickness at the fracture site (2.10 mm vs 2.16 mm, n.s). Six of the seven patients experienced significant improvement in fracture site depression, with four displaying a complete fracture reduction and two displaying a significant reduction. The degree of fracture reduction was modestly related to the depth of fracture, with the two deepest fractures failing to achieve full reduction. Age appeared to be related to fracture reduction, with the lowest reduction observed in one of the oldest patients in this sample. No complications were observed in any patient other than temporary mild swelling at the suction site, and no re-treatment or surgery for the fractures was required.

**Conclusion:**

Vacuum-assisted elevation of ping-pong skull fractures is a safe and effective noninvasive treatment option for infants that can be used under certain circumstances. The procedure can be done safely at the bedside and is a relatively quick procedure. It avoids the need for open surgical intervention, anesthesia, or hospital admission, and can lead to excellent outcomes.

## Introduction

Depressed skull fractures in infants, colloquially referred to as “ping-pong” fractures, stem from a lack of ossification in newborn skulls coupled with pressure that causes the calvarial bones to indent [1, 2]. The most common sites for ping-pong fractures to occur are the parietal and frontal bones [3]. Sutures found between the cranial bones are flexible during the early years of postnatal development, and these sites serve as the tensile points which allow the parietal or frontal bones to absorb trauma, leading to a depression within the skull [4].

Traditional treatments for ping pong fractures include surgical repair or observation for spontaneous improvement over time. Recently, vacuum-assisted elevation has been described as a viable treatment option [5–7]. Multiple studies have shown that the following scenarios may necessitate surgical repair: the presence of a foreign body, debris, or infected tissue in a wound, cerebrospinal fluid leak, presence of a hematoma, increased intracranial pressure, neurological impairment, esthetic considerations, and presence of a hygroma [8]. Though presentations such as these may necessitate surgical repair, it is desirable to avoid surgical intervention whenever possible due to the inherent potential risks associated with opening the cranium and the use of general anesthesia, particularly in neonates and infants [9].

Treatment of ping-pong fractures is dictated by the severity of the fracture itself, patient age, imaging results, and other associated injuries or complications [10]. Vacuum-assisted elevation using a negative pressure device to elevate the depression is a nonsurgical alternative that can be considered in some circumstances [10]. This method can be traced back as early as the 1970s when some providers used suction tools such as  manual breast pumps, obstetrical vacuum extractors, and pediatric CPR masks connected to a 50-mL syringe [9, 11–16].

There is a limited number of publications reviewing the outcomes of patients with depressed skull fractures corrected by suction-assisted elevation [8, 10, 15]. In this report, we review the outcomes of seven pediatric patients with ping pong fractures treated at our institution with suction elevation.

## Methods

### Participants

A retrospective chart review was conducted on all ping pong fractures treated at our institution with suction-assisted elevation between 2021 and 2022. Ping pong fractures were identified by querying our hospital’s electronic medical records database for the American Medical Association’s CPT procedure code 62000, *Repair Procedures on the Skull, Meninges, and Brain*. We identified 13 cases of young children treated for ping pong fractures, of whom six were excluded because they were treated with surgical intervention. The remaining seven cases were confirmed to have been treated using suction-assisted elevation and are included in this report. Notably, of the six excluded patients, the majority (four) were toddlers 12–38 months of age (one other child was 8 months old, and one was a neonate).

### Skull measurements

Fracture depth was obtained utilizing CT scan bone window images by measuring from the outer table of the skull to the inner table at the depth of the fracture.

Bone thickness at the depression site was measured using the ruler tool on the CT scan at three locations—at two opposing edges and the center of the depression. Mean bone thickness was calculated from these measurements.

### Suction elevation procedure

**Fig. 1:**
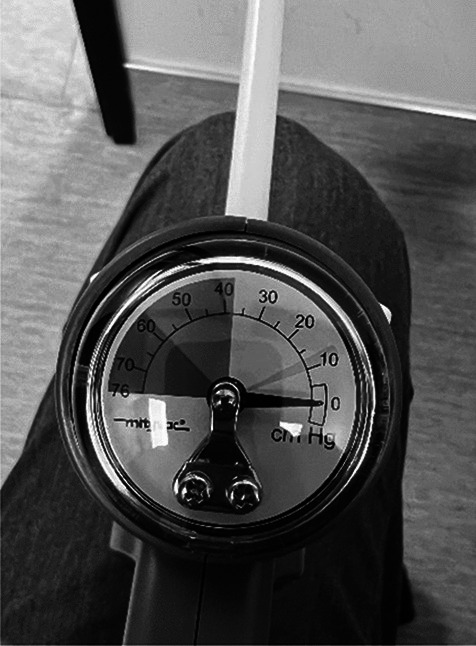
MityVac Mystic II negative pressure gauge with ideal range between 38 and 58 cm Hg and a maximum pressure of 76 cm Hg

Suction elevation was performed at the patient’s bedside in the Emergency Department (3), NICU (2), obstetric floor (1), or PICU (1) without the need for sedation or general anesthesia. Before each procedure, treatment options and risks were discussed with the parents and written informed consent was obtained. A MityVac Mystic II obstetric vacuum device was utilized. This device is commonly used for vacuum-assisted deliveries of newborns (Fig. 1). The suction site was prepared as needed (including wetting or shaving the hair if present at the fracture site) to ensure that proper suction could be achieved and maintained throughout the procedure. All patients received cardiac monitoring throughout the procedure.

The MityVac suction cup was gently placed on the child’s head directly over the visible depression of the fracture, making sure to maintain good contact between the suction cup and the scalp. The pressure on the MityVac was increased gradually, without surpassing 58 cm Hg per manufacturer recommendations, and held for several seconds, and then the suction was released. The vacuum technique was repeated if needed. While there could be some temporary soft tissue swelling immediately after the suction procedure, one could generally gauge if the skull fracture had been adequately elevated. The procedure could be repeated if needed, but the number of attempts is generally limited by bruising that can occur on the scalp, and the procedure should be stopped if there is significant scalp bruising.

### Post-procedure measurements

Following the suction elevation procedure, a fracture reduction score was assigned based on post-procedure imaging and/or the surgeon’s clinical notes. The success of fracture reduction was scored on a 4-point scale as follows, with higher scores indicating a more complete reduction: 1, unchanged; 2, mild fracture elevation; 3, significant elevation; and 4, complete elevation.

Length of hospitalization ranged from 1 to 3 days. All patients followed up with the neurosurgeon in the outpatient clinic within 2 weeks following the elevation procedure, and longer as indicated depending on the status of the elevation.

## Results

Table 1 shows the patient characteristics and injury details for the patients in this group. There were seven patients (four males, three females). Three were neonates; the four other patients ranged from 5.5 to 8.7 months of age (mean age 7 months). Most patients were treated within 1–3 days of the occurrence of the fracture, with the exception of one preterm infant for whom treatment needed to be delayed for 3 weeks postpartum. Mechanisms of injury included birth-related trauma (43%) and falls from a short height of about 3 ft or less (57%). Notably, all four of the fall victims were reported to have fallen off a bed. There was no suspicion of nonaccidental trauma for any of the patients.

**Table 1:** Patient demographics and injury details

Patient demographics					Imaging findings		
Patient	Age at injury (days)	Sex	Mechanism of injury	Time from injury to treatment (days)	Fracture depth (mm)	Average bone thickness at fracture site^a^ (mm)	Fracture location
1	0	F	Birth trauma	< 1	16.00	N/A	R parietal
2	0	M	Birth trauma	21*	9.30	2.29	R parietal
3	0	M	Birth trauma	3 days	8.29	1.90	L parietal
4	260	F	Fall	< 1 day	6.25	2.37	L parietal
5	164	M	Fall	< 1 day	5.59	2.12	L parietal
6	195	M	Fall	< 1 day	5.43	1.83	R parietal
7	205	F	Fall	< 1 day	3.89	2.30	R parietal

*N/A* imaging data not available, imaging was completed at outside hospital

^a^Average derived using measurements from the center of fracture and opposing adjacent sides

*Patient was born at 30 weeks gestation and elevation could not be done until 21 days after birth

All fractures were sustained to the parietal bone (57% right, 43% left). Fracture depth ranged from 3.89 to 16 mm. A Mann–Whitney *U*-test was used to compare mean fracture depth for patients whose skull fractures were the result of birth trauma vs accidental fall. Fracture depth for the three patients who had sustained birth trauma (11.2 mm) was deeper than that of the four patients who had sustained falls from a short distance (5.29 mm) (*p* < 0.001). Bone thickness at the site of the fracture ranged from 1.83 to 2.37 mm. There was no statistically significant difference in average bone thickness at the fracture site between the two groups (mean for birth trauma group = 2.10 mm, mean for fall group = 2.16 mm, n.s.).

Table 2 shows the details of the vacuum elevation procedures and the results for each patient. Four patients (57%) underwent one attempt at vacuum elevation lasting no more than 5 seconds, two patients (29%) underwent two attempts lasting no more than 5 seconds each, and one patient (14%) underwent six attempts lasting no more than 10 seconds each. Post-procedure outcomes for all patients indicated no associated complications. None required re-treatment or surgery for the fractures.

**Table 2:** Vacuum elevation procedure details and results

Patient	Hair prep	Suction (cm HG)	Number of attempts	Total length of procedure (sec)	Fracture reduction^a^	Admitted?	Length of hospitalization (days)
1	N/A	< 40	1	5	3	Yes	*
2	N/A	< 58	2	10	3	Yes	*
3	N/A	< 58	2	10	4	Yes	3
4	Clean, wet	< 58	1	5	4	Yes	2
5	None	35	1	5	4	Yes	1
6	Shave	< 58	1	5	4	Yes	2
7	None	30	6	60	2	No	N/A

*Patient was already hospitalized for other reasons

^a^Fracture reduction score: 1 unchanged, 2 mild fracture elevation, 3 significant elevation, 4 complete elevation

Most fractures showed immediate visible improvement following the procedure; however, the degree of fracture reduction could not be measured immediately due to the onset of scalp swelling caused by the suction procedure itself. All but one of the seven patients experienced a significant visual improvement in fracture site depression, with four (57%) displaying a complete fracture reduction (reduction score of 4) and two displaying a significant reduction (fracture reduction score of 3). Four of these six patients underwent Haste MRI imaging during follow-up examination to confirm fracture reduction and patient status. Of the other two patients, one had a complete fracture reduction at the time of the procedure by clinical exam and was noted to appear normocephalic at the 2-week follow-up clinic visit, so follow-up imaging was deferred. The other patient was followed by a clinical exam and had a transfontanelle ultrasound at the 2-week follow-up visit. None of the patients had intracranial complications.

**Fig. 2:**
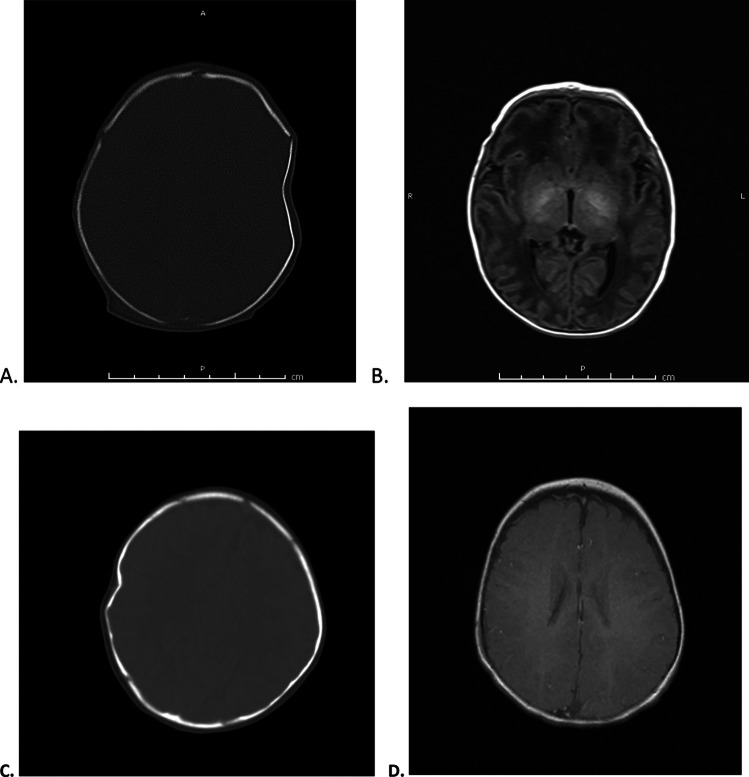
**A** Imaging of infant skull taken upon presentation for ping pong skull fracture with a 8.29-mm depression; **B** full fracture reduction following vacuum elevation; **C** imaging of infant skull taken upon presentation for ping pong skull fracture with a 5.43-mm depression; and **(D)** full fracture reduction following vacuum elevation

One of the seven cases (14.2%) displayed only a mild reduction of the fracture (reduction score of 2) as observed immediately following the procedure and before being discharged from the hospital. Figure 2 shows the pre- and post-procedure imaging of two patients for whom full reduction of the skull fracture depression was achieved via vacuum elevation.

The degree of fracture reduction was modestly related to the depth of the original fracture, with the two deepest fractures failing to achieve full reduction (reduction score of 3). The exception to this pattern was a nearly 7-month-old patient with the most shallow fracture (depth of 3.89 mm), six vacuum elevation attempts (maximum pressure of 30 cm Hg, below the range of 38-58 cm Hg), and only mild fracture reduction (score of 2). This patient was one of the older patients in the sample, thus underscoring the likelihood that suction elevation has limited efficacy in older infants and toddlers.

The four patients who experienced complete fracture reduction did not show any residual signs of skull depression. Of the three patients who did not have complete reduction immediately, all were seen over the course of the next several months, and complete reduction was noted by 2, 4, and 7 months post-fracture.

## Discussion

Our experience with the MityVac Mystic II Delivery System highlights the efficacy of suction elevation for the treatment of ping-pong skull fractures. The MityVac Mystic II is a common obstetric handheld device that can be found in most hospitals or easily procured. It is designed to be placed on the heads of newborn infants. The small scale, portability, and design of the system allow surgeons to deploy this tool at the bedside.

In contrast to other reports of vacuum-assisted skull fracture repair [10], in this paper we report that this procedure can be done safely without anesthesia. More recently, Minghinelli and colleagues [17] reported that, similar to our own findings, the vacuum elevation of a ping-pong fracture could be done safely without the use of anesthesia. The use of anesthesia in neonates and infants carries its own potential risk factors, including deleterious effects on neurocognition and physiological effects such as hypotension, hypocapnia, and hyper-/hypoxia [9].

Vacuum-assisted elevation is also less costly than open surgery. The lack of anesthesia or operating room time, as well as quicker recovery and potentially fewer hospital days, leads to reduced overall cost of treatment.

We found that fractures resulting from birth trauma tended to be deeper than those resulting from falls. The three patients in our sample who experienced skull fractures at the time of delivery had fracture depths ranging from 8 to 16 mm. As reported in the literature, skull fractures at the time of birth can occur as a result of the head pressing into the maternal pelvis or from the use of surgical instruments to deliver the neonate [7]. However, we did not find any literature reporting that skull fractures caused by birth trauma tend to be deeper than those resulting from falls. Nevertheless, in our sample vacuum elevation was effective regardless of the manner of fracture or degree of severity, with six of the 7 patients in this sample experiencing significant reduction of the fracture at the time of the procedure.

Bone thickness and location of the fracture did not seem to predict whether vacuum elevation would be successful in this sample. All seven cases reported here were parietal skull fractures. Contrary to findings reported in the literature showing a relation between decreased bone thickness and fracture reduction success [10, 16], we did not find such a relation between bone thickness and success of fracture reduction, possibly because bone thickness varied little among patients in this group.

The depth of the fracture seemed to be an important factor in the success of vacuum elevation, as others have reported [8]. There was a trend toward the deepest fractures in this group requiring a second vacuum attempt. In addition, the two deepest fractures had slightly less complete reduction compared to most of the rest of the fractures. However, all but one patient had a significant reduction, and most had complete reductions.

An interesting finding was that all four fractures resulting from falls in this sample occurred in the same way, namely by falling off a bed at home (all were 6–9 months old at the time of injury). This finding is consistent with research showing that the most common mechanism of infant falls from a short vertical distance is rolling off a bed [18]. This reinforces that pediatricians should discuss bed safety practices with parents as part of routine well-child examinations in the first year of life. In our sample, there was no suspicion of nonaccidental trauma in any of these patients. Nonetheless, hospital personnel should be alert for signs of nonaccidental trauma and take appropriate actions when indicated [19].

In our institution, older infants and toddlers were more likely to be treated with surgical repair (not included in this review). As reported in the literature, vacuum elevation is of limited utility in older infants [10]. The limited efficacy of vacuum elevation in older infants may be due in part to the size and thickness of the skull and the inability to achieve adequate suction with a device intended to fit the typical newborn skull. Indeed, the one patient in this sample for whom the lowest reduction was achieved was among the oldest infants in this sample.

Our results suggest that vacuum elevation is a good treatment option in young infants that avoids the inherent risks associated with anesthesia or open surgical repair. The procedure is able to be done at the bedside, with minimal patient discomfort and quick recovery.

## Limitations

This study had a limited sample size and was a retrospective review. Three patients did not have post-procedure imaging available and were followed clinically to confirm the degree of fracture reduction.

## Conclusions

Vacuum-assisted elevation for the treatment of ping-pong depressed skull fractures in infants is safe and effective. It provides several benefits, including avoiding open surgical intervention, short procedure time, no blood loss, decreased cost, decreased pain, avoiding anesthesia, and short hospital stay. It should be considered as a treatment alternative in young infants with ping-pong skull fractures.

## Data Availability

The data that support the findings of this study are not openly available due to protection of patient confidentiality and are available from the second author (VDA) upon reasonable request. Data are located in controlled access data storage at Children's Hospital of Orange County.
